# EVICAN—a balanced dataset for algorithm development in cell and nucleus segmentation

**DOI:** 10.1093/bioinformatics/btaa225

**Published:** 2020-04-01

**Authors:** Mischa Schwendy, Ronald E Unger, Sapun H Parekh

**Affiliations:** b1 Max Planck Institute for Polymer Research, Mainz 55128, Germany; b2 Institute of Pathology, Universitätsmedizin-Mainz, Mainz 55131, Germany; b3 Department of Biomedical Engineering, University of Texas at Austin, Austin, TX 78712, USA

## Abstract

**Motivation:**

Deep learning use for quantitative image analysis is exponentially increasing. However, training accurate, widely deployable deep learning algorithms requires a plethora of annotated (ground truth) data. Image collections must contain not only thousands of images to provide sufficient example objects (i.e. cells), but also contain an adequate degree of image heterogeneity.

**Results:**

We present a new dataset, EVICAN—Expert visual cell annotation, comprising partially annotated grayscale images of 30 different cell lines from multiple microscopes, contrast mechanisms and magnifications that is readily usable as training data for computer vision applications. With 4600 images and ∼26 000 segmented cells, our collection offers an unparalleled heterogeneous training dataset for cell biology deep learning application development.

**Availability and implementation:**

The dataset is freely available (https://edmond.mpdl.mpg.de/imeji/collection/l45s16atmi6Aa4sI?q=). Using a Mask R-CNN implementation, we demonstrate automated segmentation of cells and nuclei from brightfield images with a mean average precision of 61.6 % at a Jaccard Index above 0.5.

## 1 Introduction

In recent years, microscopy has seen major advancements in both the optical and automation performance. The rise of automation in the industry: both in acquisition and analysis, has turned microscopes into powerful high-content screening systems. Researchers are now in need of rapid and accurate analyses to infer quantitative measures from the ever-increasing amount of imaging data. The two most essential steps in deriving quantitative data from images are segmentation and classification. Segmentation is the process of finding the outlines of objects within an image while classification identifies the object by assigning a class label (e.g. ‘Nucleus’ or ‘Cell’). In cell biology applications of microscopy, cellular and subcellular entities can be segmented and classified to enable single-cell analyses and relate measured quantities, such as cell shape or intensity of a fluorescent molecule within cells, in response to specific treatments.

Traditionally, image processing in cell biology applications of microscopy have been based on fluorescent staining of cells (Schwendy *et al.*, 2019; Wählby *et al.*, 2002). Fluorescent staining offers two primary benefits: firstly, classification can be performed based on the compartmental or molecular specificity of different fluorophores (e.g. the fluorescence channel for membrane-stain corresponds to the ‘cell’-class, while the channel for a nucleus-stain corresponds to the ‘nucleus’-class). Secondly, image contrast in stained images arises from the abundance of fluorophores that accumulate in specific compartments against a zero background in the ideal case, which facilitates object segmentation. In contrast, cell segmentation and classification in (unstained) brightfield (BF) or phase-contrast (PhC) images is not trivial, as images exhibit a highly irregular appearance, non-specific contrast from cells and a non-zero background. While fluorescence staining is immensely powerful and convenient for cellular analyses and identification, it is nevertheless often associated with non-ideal requirements, such as the need for cell fixation and permeabilization and introduction of exogenous molecules, rendering it difficult to observe processes in native, live-cell experiments. Even if a dye does not require a sample preparation that results in cell death, it can introduce perturbations into the system that alter the experimental outcome (Erba *et al.*, 1988; Knight *et al.*, 2003; Wiedenmann *et al.*, 2009). A segmentation and classification algorithm that performs robustly on unstained cell images would, therefore, be beneficial economically (reducing costs for fluorescent dyes and reducing hands-on work) and scientifically (possibility of unperturbed, quantitiative live-cell imaging).

A promising potential solution lies in using ‘big data’ in conjunction with deep learning analyses. Specifically, convolutional neural networks (CNNs) offer the ability to train an algorithm to identify complex patterns and classify objects within images (Krizhevsky *et al.*, 2012; LeCun *et al.*, 1998). In recent years, CNNs have advanced beyond classifying image content to also localizing (Girshick, 2015; Girshick *et al.*, 2014; Ren *et al.*, 2017), and finally segmenting, objects within an image (He *et al.*, 2017). The new possibilities provided by CNNs have influenced a broad range of engineering and scientific fields, such as autonomous driving (Bojarski *et al.*, 2016), face recognition (Schroff and Philbin, 2015) and cancer detection (Esteva *et al.*, 2017). CNNs have also begun to have an impact in cell biology as demonstrated by two very recent deep learning approaches to predict cellular architecture using a trained CNN. Christiansen *et al.* (2018) trained a deep neural network for *in silico* prediction of fluorescent labels, such as nucleus-, membrane- and axon-staining. Their algorithm used several BF or PhC microscopic slices of varying z-depth to generate a predicted maximum-intensity projected fluorescent image (single plane). Another approach by Ounkomol *et al.* (2018) achieved 3D fluorescent label prediction from 3D confocal stacks. Prediction of fluorescent labels is a powerful tool in cell microscopy but is not the same a single-cell quantification within images, for which appropriate segmentation steps are required. CNNs have also been applied for cell segmentation as first demonstrated by Ronneberger *et al.* (2015).

A major drawback of CNNs, however, is their need for massive amounts of annotated data. Annotated image collections for everyday-scene analysis exceed 100 000, or even 1 000 000 images (Deng *et al.*, 2009). For instance, the Common objects in context (COCO) dataset (Lin *et al.*, 2014), a popular collection of images for the training of segmentation algorithms, contains more than 330 000 images with more than 2.5 million labeled instances of 91 different classes. Such large collections are needed to provide the CNN architecture with sufficient exemplary structures to create appropriate filters and thereby identify objects in images correctly. An adequate dataset is not only a question of size but also of heterogeneity, as a dataset needs to capture (instrument- and object-specific) image and shape variations to it to be generally applicable. In cell biology applications of microscopy, images vary by factors, such as light intensity, magnification, contrast, and uneven illumination. Additionally, objects within the images (cells, nuclei, granules, etc.) are highly heterogeneous entities that adopt various forms and sizes, e.g. cell shape can change from the ‘fried egg’ appearance to an elongated form under various stimuli. Low image heterogeneity in a dataset, e.g. by training on a single imaging setup can result in a need to retrain the resulting algorithm before usage with other imaging equipment (De Fauw *et al.*, 2018).

To our knowledge, most databases used for CNN approaches for cell biology applications are limited in terms of image heterogeneity such that images often come from a single setup, using the same objective, and exhibit limited variation in terms of cell types. To increase the ability for computer vision experts to develop image segmentation and quantitative image processing using CNNs that are applicable to more types of cells and microscopy conditions, we assembled a collection of more than 4600 images of 30 cell lines, acquired on 4 separate microscopy setups in three different laboratories with 9 different objectives having magnifications ranging from 10× to 40×. Cellular outlines and nuclei in our dataset were segmented manually by cell culture experts against fluorescently stained images. To our knowledge, this represents the first freely available, large-scale segmented dataset with more than 20 cell lines in the cell culture sector. With this new dataset, we hope to help close the gap between cell biologists and computer scientists, as it provides access to biological data specifically prepared for training of computer vision algorithms. As a proof-of-principle, we also trained a segmentation and classification algorithm on our dataset and achieved an average precision (AP) of 61.6% for intersection over union (IoU) scores above 0.5.

## 2 Materials and methods

### 2.1 Cell culture

All cells were maintained at 37°C, 90% relative humidity and 10 U/ml Penicillin/Streptomycin (Gibco) added to the respective medium. A complete list of used cell lines together with media and indicated culture supplements, such as fetal calf serum (FCS) and non-essential amino acids (NEAA), is given in Table 1. At 24 h before imaging, cells were seeded into 96-well plates (Cellstar, Gibco; Screenstar, Greiner Bio-One) at 30%, 50% and 100% confluency. After incubation, cells were fixed with 4% para-formaldehyde in phosphate-buffered saline (PBS) for 10 min. Prior to imaging, cell membranes were stained with 0.01% CellMask Orange (Thermo Fisher Scientific) in PBS, and nuclei were stained with 1 µg/ml DAPI (Thermo Fisher Scientific) in PBS for 20 min at 37°C. Cells were subsequently washed with PBS three times prior to imaging.

**Table 1.** Cell lines represented in the EVICAN dataset

No	Cell line	Species	Tissue	Type	Medium	FCS (% in medium)	NEAA added	Code
**1**	Colo 320	Human	Colon	Colon adenocarcinoma	RPMI	10	x	ACC 144 (DSMZ)
**2**	SW-480	Human	Colon	Colorectal adenocarcinoma	RPMI	10	x	CCL-228 (ATCC)
**3**	HT-29	Human	Colon	Colorectal adenocarcinoma	RPMI	10	x	HTB-38 (ATCC)
**4**	Caco-2	Human	Colon	Colorectal adenocarcinoma	EMEM	20	a	HTB-37 (ATCC)
**5**	DLD-1	Human	Colon	Colorectal adenocarcinoma	RPMI	10	x	CCL-21 (ATCC)
**6**	HCT116	Human	Colon	Colorectal carcinoma	RPMI	10	x	CRL-247 (ATCC)
**7**	RKO	Human	Colon	Colon carcinoma	EMEM	20	a	CRL-2577 (ATCC)
**8**	T47D	Human	Mammary gland	Ductal carcinoma	RPMI	10	x	HTB-133 (ATCC)
**9**	SK-BR-3	Human	Mammary gland (derived from pleural effusion)	Adenocarcinoma	RPMI	10	x	HTB-30 (ATCC)
**10**	MDA-MB-231	Human	Mammary gland (derived from pleural effusion)	Adenocarcinoma	RPMI	10	x	HTB-26 (ATCC)
**11**	MCF-7	Human	Mammary gland	Adenosarcoma	RPMI	10	x	HTB-22 (ATCC)
**12**	786-O	Human	Kidney	Renal cell adenocarcinoma	RPMI	10	x	CRL-1932 (ATCC)
**13**	769p	Human	Kidney	Renal cell adenocarcinoma	RPMI	10	x	CRL-1933 (ATCC)
**14**	ACHN	Human	Kidney	Renal cell adenocarcinoma	EMEM	20	a	CRL-1611 (ATCC)
**15**	CAKI-2	Human	Kidney	Clear-cell carcinoma	RPMI	10	x	HTB-47 (ATCC)
**16**	PC-3	Human	Prostate	Adenocarcinoma	50/50 RPMI/F12	10	x	CRL-1435(ATCC)
**17**	LNCaP	Human	Prostate	Carcinoma	RPMI	10	x	ACC 256 (DSMZ)
**18**	DU-145	Human	Prostate (derived from metastatic site in brain)	Carcinoma	RPMI	10	x	HTB-81 (ATCC)
**19**	SH-SY5Y	Human	Bone marrow neuroblastoma	Neuroblastoma	DMEM	20	x	CRL-2266 (ATCC)
**20**	MG-63	Human	Bone	Osteosarcoma	EMEM	20	a	CRL-1427 (ATCC)
**21**	HeLa	Human	Cervix	Adenocarcinoma	DMEM	10	x	CCL-2 (ATCC)
**22**	HT-1080	Human	Connective tissue	Fibrosarcoma	DMEM	10	x	CCL-121 (ATCC)
**23**	NIH/3T3	Mouse	Embryo	Fibroblast	DMEM	10	x	CRL-1658(ATCC)
**24**	RAW 264.7	Mouse	Ascites (Abelson murine leukemia virus-induced tumor)	Macrophage	DMEM	10	x	TIB-71 (ATCC)
**25**	HEL 299	Human	Lung	Fibroblast	DMEM	10	x	CCL-137 (ATCC)
**26**	FaDu	Human	Pharynx	Squamous cell carcinoma	DMEM	10	x	HTB-43 (ATCC)
**27**	MCC26	Human	Skin	Merkel carcinoma from skin	DMEM	10	x	10092304 (Sigma Aldrich)
**28**	C2C12	Mouse	Muscle	Myoblast	RPMI	10	x	CRL-1772 (ATCC)
**29**	CHO-K1	Hamster	Ovary	Epithelium	F12	10	x	CCL-61 (ATCC)
**30**	hMSC	Human	Bone marrow	Mesenchymal stem cells	DMEM	10	x	PT-2501 (Lonza)

### 2.2 Imaging

#### 2.2.1 Image acquisition

Image acquisition was performed on four different microscope setups: Opera Phenix (Perkin Elmer), AF7000 (Leica), IX81 (Olympus) and Biorevo BZ-9000 (Keyence). Table 2 summarizes the microscopy platforms as well as objectives used in this work.

**Table 2.** Microscopes and objectives used for image acquisition

Microscope	Objective	Contrast mode
Opera Phenix (Perkin Elmer)	10×/0.3 (air)	BF
	20×/0.4 (air)	BF
	40×/1.1 (water)	BF
IX81 (Olympus)	10×/0.3 (air)	PhC
	20×/0.4 (air)	PhC
AF 7000 (Leica)	10×/0.3 (air)	PhC
	20×/0.4 (air)	PhC
Biorevo BZ-9000 (Keyence)	10×/0.3 (air)	PhC
	20×/0.45 (air)	PhC

#### 2.2.2 Image annotation and dataset assembly

**Fig. 1.**
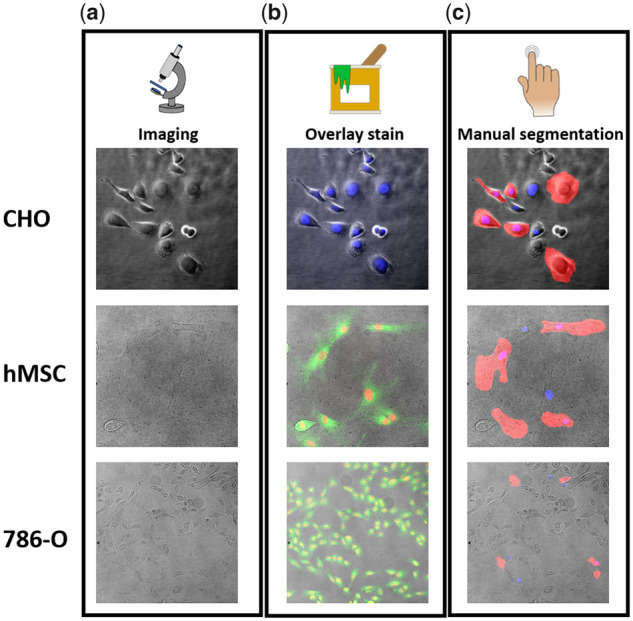
Dataset preparation pipeline. (**a**) Acquisition of BF and PhC microscopy images from various microscopes at different magnifications. (**b**) Overlay images of fluorescent nuclei and membrane, where available. (**c**) Manual segmentation of cell bodies (red) and nuclei (blue) were saved as binary masks and transformed into the COCO segmentation format. (Color version of this figure is available at *Bioinformatics* online.)

BF or PhC microscopy images were merged with region-matched fluorescence images of the nucleus and membrane to facilitate recognition of nuclei and cell borders using FIJI**^®^** (Schindelin *et al.*, 2012). Cell culture experts and supervised personnel annotated 3–10 cells (on average 5.70 per image) and nuclei (on average 5.72 per image) within each image via the process depicted in Figure 1. To reduce human bias, cells and nuclei for annotation were picked at random by one of seven annotators. Need for corrections of the segmentation masks by the lead author was extremely rare (estimated at <0.5%). For more detailed information on comparability between trained and untrained annotators, we refer the reader to work by Hughes *et al.* (2018). The final dataset contained three subsets:

1. Training dataset: 4464 images [3714 partially annotated; 750 background (blank) images; 42 317 instances]
2. Validation dataset: 1176 images [926 partially annotated; 250 background (blank) images; 10 642 instances]
3. Evaluation dataset: 98 images (fully annotated; 3222 instances).

In total, 52 959 instances were segmented in the 4640 partially annotated images of the training and validation datasets. All annotations were exported as JavaScript Object Notation (JSON) document with IDs referring to the BF or PhC version of the original image, segmentations as x, y-polygons and category-IDs indicating cellular entity or nucleus. The export format was chosen to fit COCO annotation style to ensure maximal accessibility for modern machine-learning training. The dataset is available under a CC-BY license to allow far-ranging use. We encourage scientists to more annotations to the training and validation subsets and therefore supplied segmentation masks and raw images. The complete collection of dataset and annotation documents is provided under https://edmond.mpdl.mpg.de/imeji/collection/l45s16atmi6Aa4sI?q=.

### 2.3 Convolution neural network training and evaluation

#### 2.3.1 Classifier training

Training our detection and segmentation algorithm was performed on a Mask R-CNN implementation, previously released under an MIT license by Matterport Inc. (Mask R-CNN implementation by Matterport Inc., 2018, https://github.com/matterport/Mask_RCNN). In this implementation, the Mask R-CNN approach is executed using the open-source Tensorflow and Keras libraries. As images were acquired on a variety of optical setups and with different cameras, all images were automatically adjusted to a size of 1024×024 pixels, with zero padding in cases where the raw image files were smaller by the Mask R-CNN implementation. We used a modification of the training scheme published previously by Johnson (2018). Briefly, a Resnet-101 feature pyramid network model with 101 layers organized in five stages was employed as the backbone; weights were initialized with pre-trained weights on the COCO dataset; training was performed for 52 epochs. A total of 40 epochs were used to train the so-called network heads, 8 epochs for layers 4 and above (4+), and 4 epochs for training of all layers [for more information on the network architecture, we refer the reader to He *et al*. (2016) and He *et al.* (2017)]. The learning rate for weight adjustment during training was set to an initial value of 0.001 at the start of heads, as well as 4+ layer training; this value was decreased by 50% after 20 epochs for heads training and after 4 epochs for 4+ layer training, respectively. For end-to-end training after epoch 48, we decreased the learning rate to 10% of the original value and after an additional 2 epochs, decreased it to 5% of the original value. All training steps were carried out on a desktop PC with an Intel Core i7-6700 CPU with four 3.4 GHz processors and 50 GB RAM for a full process duration of 31 days. No GPUs were used in our implementation. The code used to train the classifier is available at https://github.com/MischaSchwendy/EVICAN-MRCNN.

#### 2.3.2 Classifier segmentation evaluation

The evaluation was performed on 98 fully annotated BF and PhC images that were removed and kept separate from the training data. According to the image quality characteristics summarized in Table 3, we categorized each evaluation image into one of three difficulty classes.

**Table 3.** Quality characteristics of evaluation datasets

Evaluation dataset	Cellular appearance (in PhC or BF)	Image quality, contrast mechanism
Difficulty 1 33 images, 1084 instances	2D cell growth, few cell–cell contacts, clear-cell outlines, most nuclei visible	All cells in focus, most often PhC
Difficulty 2 33 images, 1036 instances	2D cell growth, several cell–cell contacts, most cell outlines visible, few nuclei visible	Cells minimally defocused, mixed BF and PhC
Difficulty 3 32 images, 1102 instances	Occasional 3D growth, many cell–cell contacts/ colony formations, nuclei often invisible without staining	Frequently defocused, mainly BF images

The three resulting evaluation datasets are intended to assess the capabilities of the trained classification-segmentation algorithm under varying imaging conditions. To guarantee accuracy of the ground truth masks, all annotations for quantitative comparison were generated on fluorescently stained images.

Classification performance was evaluated according to the average precision (AP) metric. Predicted instances co-localized with corresponding ground truth instances were counted as true positives when exhibiting intersection over union (IoU) scores above a certain threshold. We monitored AP at IoU thresholds above 0.5 (AP_0.5_) and 0.75 (AP_0.75_) and report averaged values over all evaluation images. Additionally, a cumulative AP was computed, where the IoU threshold was incrementally increased from 50% to 95% in 5% steps, and the precision per image averaged over each step.

## 3 Results

### 3.1 Dataset curation

We assembled a dataset consisting of more than 4600 partially segmented BF and PhC microscopy images using several different microscopy setups. Up to 10 cellular and nuclear outlines were segmented per image, respectively, with a per image average of 11.4 total instances. As depicted in Figure 1, the pipeline we established for dataset production was to overlay PhC (or BF) microscopy images (Fig. 1a) with fluorescent channels from membrane and/or nucleus staining. Regarding the images in Figure 1c, the ratio of annotated instances to true instances within the image declined with lower magnification since more cells were included in the lower magnification images. Manual annotation was executed and validated by experienced cell biologists, which—while being quite laborious—was the only way to guarantee human-level accuracy for all images.

**Fig. 2.**
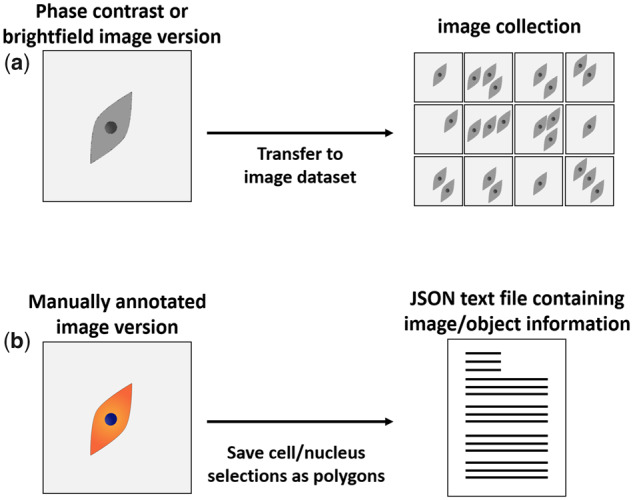
Dataset compilation. (**a**) Grayscale (PhC and BF) image versions were collected in a large-scale image collection. (**b**) Manual annotations (cell and nucleus segmentations) were transformed into polygons and saved in a JSON file. The compiled text file included information about images (ID, size and name) and objects (ID, class and polygon). All text information is referred to the grayscale image versions produced in (a)

Annotated images and corresponding grayscale versions (i.e. BF and PhC images) were compiled to a dataset consisting of a text file and an image collection in JPEG-format (Fig. 2). Manual annotations (i.e. segmentations) performed on corresponding stained overlay images were transformed into polygons (i.e. ‘X1, Y1; X2, Y2;…; Xn, Yn’) and saved together with image and object information, in a JSON text file. While annotations were performed on stained images, the information saved in the text file referred to the assembled PhC/BF image collection. Referring to the BF and PhC images for classifier training was necessary to ensure the usability of the resulting computer vision algorithms for unstained cell images after training on this dataset. Fluorescently stained images were not included in the compiled dataset.

**Fig. 3.**
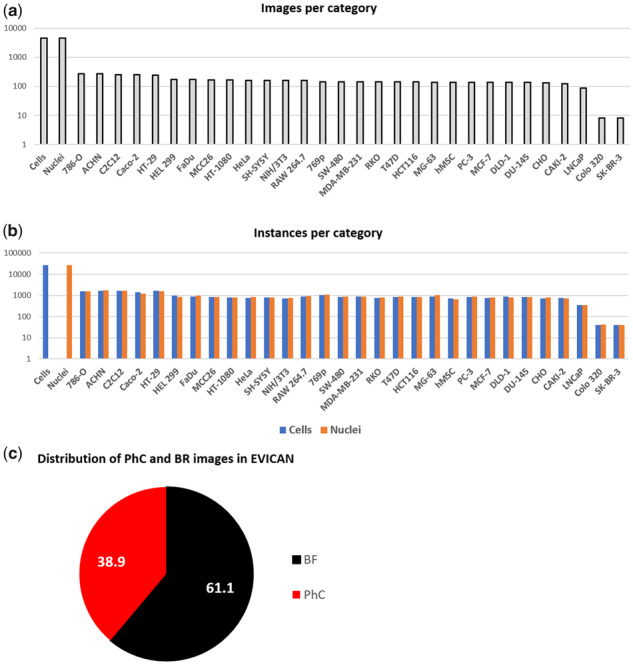
Overview of the EVICAN dataset. (**a**) Number of images per cell line, (**b**) numbers of instances per category in the dataset, and (**c**) relative number of images for BF and PhC imaging in the dataset

Figure 3a shows the number of images for each cell line, with the majority containing >100 images. We provide two datasets in the COCO annotation format: the EVICAN2-version with two classes: ‘cell’ and ‘nucleus’, and the EVICAN60-version with nuclei and cells classified for each cell line, respectively, resulting in 60 class labels. As Figure 3b shows, we achieved a highly homogeneous distribution of nucleus and cell instances across nearly all cell lines. For most classes in the EVICAN60-version, we provide ∼1000 instances; for the cell and nucleus class in EVICAN2, we exceeded 26 000 instances per class. Figure 3c shows the moderate underrepresentation of PhC images in our dataset, which as described later, contributes to less prominent feature observability due to reduced contrast in BF compared to PhC images. Additional to the COCO-format annotations, we provide all masks as binary images with format ‘imageID_cellline.jpg’ to offer freedom for developers and researcher not using COCO-like datasets. The complete collection of images, masks and annotation documents is available at: https://edmond.mpdl.mpg.de/imeji/collection/l45s16atmi6Aa4sI?q=.

### 3.2 Comparison to other segmented cell datasets

The EVICAN dataset generated above was compared to four other commonly used cell biology segmentation datasets:

1. The Allen Cell Explorer dataset [*Allen Institute for Cell Science, Allen Cell Explorer data,* 2019, https://www.allencell.org/data-downloading.html#DownloadImageData); Roberts *et al.*, 2017]: a collection of ∼39 000 manually curated cells in ∼18 000 confocal 3D z-stacks (Personal correspondence with Nathalie Gaudreault, Associate Director Microscopy Pipeline, 2019). Transmitted light images, as well as manually curated segmentation masks for 12 cellular components, are provided.
2. The total number of PhC and DIC microscopy images of cell lines from the International Symposium on Biomedical Imaging (ISBI) cell-tracking challenges from 2014 and 2015: containing 6 time-lapse microscopy recordings, with 56 annotated frames of 3 cell types (HeLa, pancreatic stem cells and U373 cells) (Ronneberger *et al.*, 2015; Ulman *et al.*, 2017).
3. Three combined datasets of PhC images by Gurari *et al.* (2015): 151 partially segmented images of 3 cell lines (fibroblasts, rabbit smooth muscle cells and rat smooth muscle cells).
4. All DIC microscopy images of cell lines from the Broad Bioimage Benchmark Collection (BBBC) (Ljosa *et al.*, 2012): 65 fully segmented images for 2 cell lines: CHO and red blood cells.

In comparing datasets, the size, i.e. the number of images and segmented instances/cells, as well as heterogeneity was reviewed. The heterogeneity of a dataset should ideally reflect object- and instrument-specific variations to be complete. To account for object- (i.e. cell) specific variations, we assessed the number of cell lines included in a dataset, as well as cellular structures (e.g. cell, nucleus, and actin) annotated in a dataset: object variation=# cell lines×# cell structures. For instrument-specific variations, we checked for the number of different microscope models, magnifications, and contrast mechanisms used in each dataset: instrument variation=# magnifications×# microscope models×# contrast mechanisms (Table 4).

**Table 4.** Number of images and segmented cells in the five compared datasets

	EVICAN	Allen cell explorer	ISBI	Gurari	BBBC
Images	4640	∼ 18 000	56	151	65
Segmented cells	26 428	∼ 39 000	899	151	995
Cell lines	30	1	3	3	2
Cell structures	2	12	1	1	1
Magnifications	3	1	3	2	2
Microscope models	4	1	3	1	1^a^
Contrast mechanisms	2	1	2	1	1
Object variation	60	12	3	3	2
Instrument variation	24	1	18	2	2

*Note*: Highest scoring datasets for each category are highlighted in gray.

a Potentially more microscope models.

In particular, increasing object variation, i.e. ‘cell lines’ and ‘cellular structures’, is valuable for algorithm development, as it allows computer scientists to generate multiple classes. For instance, in our EVICAN2 dataset only *segmented structures* were introduced as classes, i.e. the two classes ‘nucleus’ and ‘cell’, while in the EVICAN60 dataset, each *cell line* and *segmented structures* were used to form 60 classes (nuclei and cellular outlines specific for each of the 30 cell lines).

Only the Allen Cell Explorer dataset outranks the EVICAN dataset in terms of image number (18 000 versus 4600) and number of segmented cells (39 000 versus 26 400). The ISBI image collection shows a strong instrument-specific variation with multiple objective magnifications, microscope models and contrast mechanisms. In contrast, the Allen Cell Explorer data collection provides a strong object-specific variation, due to the multitude of segmented cellular structures that are available as 3D masks. However, none of the compared image collections achieves a heterogeneity greater than our EVICAN dataset, which offers large image and instance numbers combined with a balanced object and instrument variation. The comparison datasets either provide only a limited number of images (ISBI, Gurari and BBBC) or show limited instrument-specific heterogeneity (Allen Cell Explorer). Limited data and variation within a dataset reduce the robustness of trained algorithms for general use outside of, e.g. the specific imaging system or specific cell lines used in training.

### 3.3 Dataset usage in segmentation analysis

#### 3.3.1 Classifier training

As a proof-of-principle demonstration that the EVICAN dataset is useful for deep learning applications, we used the EVICAN2 version to train a deep learning classifier using a modified version of Matterport Inc.’s implementation of Mask R-CNN for image segmentation and object classification. To reduce the influence of unannotated cells on the background class, we prepared our dataset by Gaussian blurring (sigma =30 pixels) everything except for the annotated instances plus an extra 10-pixel border around their outlines. The blurred content in our images was our solution to using partially annotated images and minimizing the incorrect training of the classifier to consider non-segmented cells as a requirement for segmented cells. Additionally, several hundred unblurred background images (having no cells) were included in the training and validation dataset to allow for an appropriate training of the background class. The Mask R-CNN algorithm was then trained as described in Section 2. The trained classifier produced an algorithm for cellular and nuclear detection based on both BF and PhC images.

#### 3.3.2 Classifier evaluation

**Fig. 4.**
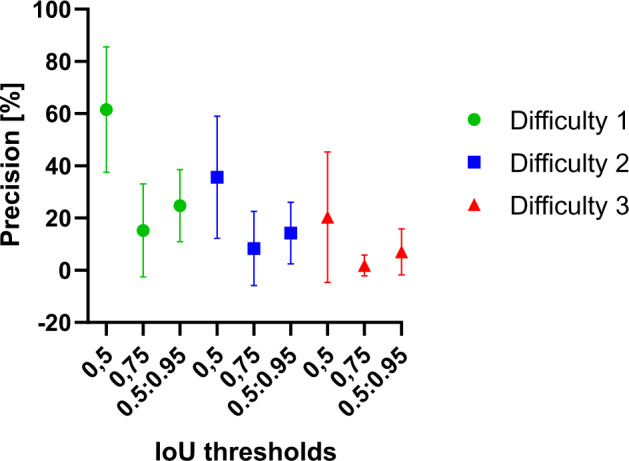
AP of our trained algorithm on EVICAN2 evaluation images of three different difficulty levels. All AP values decrease with increasing difficulty and increasing IoU thresholds. Error bars are shown as standard deviation. AP values decrease with increasing difficulty level

We tested our trained algorithm on microscopy image evaluation datasets categorized in three classes of rising difficulty level. AP was computed at IoU thresholds above 0.5 (AP_0.5_), above 0.75 (AP_0.75_), and averaged over thresholds rising from AP = 0.5 to 0.95 in 0.05 steps. All values were averaged over all images within the respective evaluation dataset. Figure 4 shows that with rising difficulty level of the evaluation data and with increased IoU thresholds, AP values decreased, as expected.

**Fig. 5.**
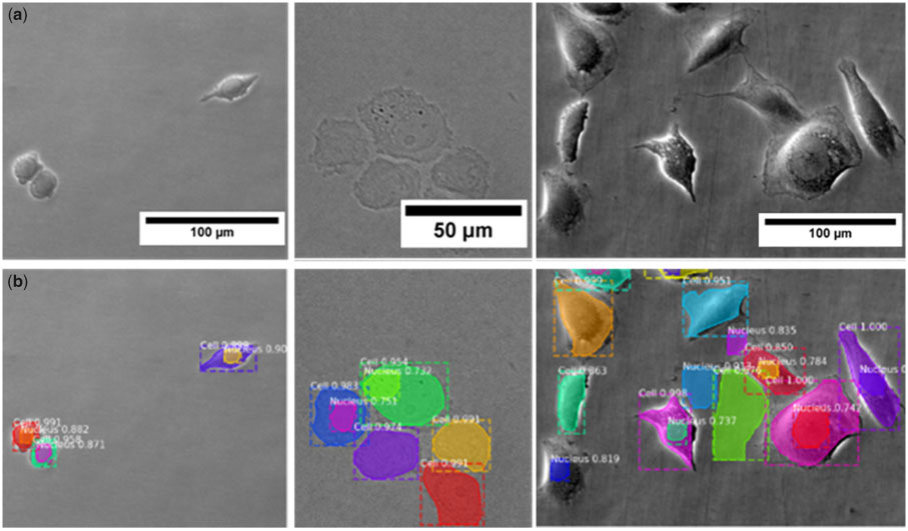
EVICAN2-trained classifier performance for grayscale image segmentation. Input (**a**) and output (**b**) example images for our algorithm. Varying colors indicate individual cell or nucleus segmentations, values (white) denote confidence of each detection (maximum = 1.0; all values above 0.7). Left: SW480 cells, imaged in PhC mode (20× objective), 100% correctly detected cells; middle: PC3 cells, imaged in BF mode (40× objective), nuclei not or incorrectly detected; right: CHO cells, imaged in PhC mode (20× objective), several false-positive detections. (Color version of this figure is available at *Bioinformatics* online.)

When assessing the lowest difficulty evaluation dataset, we achieved an AP_0.5_ of 0.61. The decreasing AP with increasing IoU thresholds (i.e. scores of AP_0.75_ and AP) indicated that a majority of positive detections in AP_0.5_ was based on IoU values below 0.75. With the combined annotation of cell bodies and nuclei in one dataset, we could also show that it was possible to detect cells and subcellular features mutually in one step. For qualitative assessment, Figure 5a shows exemplary input and output images for our algorithm. It is apparent that the algorithm produces better results on images with higher contrast (i.e. in PhC images, see Fig. 5, left and right columns).

BF images, even with high magnification, often result in incorrect detections or missed cells/nuclei (see Fig. 5, middle column). While other datasets include colored or stained images, EVICAN relies solely on grayscale images, thereby limiting feature dimensionality.

## 4 Discussion

The EVICAN dataset provides a large-scale, multi-class, manually annotated and segmented, mixed BF and PhC microscopy image collection covering a broad range of cell lines (30 adherent cell lines). Training computer vision algorithms on our dataset should enable computer scientists to produce faster and more accurate, and more broadly usable, cell image segmentation and characterization tools using unstained images. This capability has the potential to strongly increase the ability of simple light microscopes to serve as quantitative instruments in cell biology labs. Machine-learning algorithms have been applied before to microscopy images, in part with remarkable success, such as work by Christiansen *et al.* (2018) and Ounkomol *et al.* (2018) who predicted fluorescent labels from transmitted light images. However, image processing is still far behind the technology for image acquisition. Despite the massive application of computer vision in other data-intensive sectors like face recognition (Schroff and Philbin, 2015), progress in applying computer vision in cell microscopy has been comparatively slow. We believe that this slow progress is due to the two decoupled sectors: computer scientists usually have no access to a biolab with adequate image acquisition machinery and most biologists lack the knowledge and skills to create or retrain computer vision algorithms. Image collections like the ISBI cell-tracking challenge datasets offer relatively heterogeneous datasets but lack appropriate image numbers. In contrast, the Allen Cell Explorer dataset offers gigantic numbers of images and instances but lacks image heterogeneity. The scope of the Allen Cell Explorer dataset is to provide confocal z-stacks with a multitude of manually curated cellular structures that allow training of machine-learning algorithms to detect subcellular entities. The limited heterogeneity is a result of the focus on high-throughput experiments on a single-cell line (human-induced pluripotent stem cells), using identical microscope models, and providing the image data from a single objective magnification (100×). We note that an extensively annotated fluorescent dataset of nuclei was recently released by Carpenter and colleagues with 23 165 segmented nuclei as well as an evaluation of deep learning strategies (Caicedo *et al.*, 2019).

The limitations of current state-of-the-art image datasets for cell microscopy leave room for a balanced dataset of sufficient size. We hope to fill this gap by providing computer scientists (and other algorithm developers) with our image collection. We provide two editions of our dataset: EVICAN2 with classes ‘nucleus’ and ‘cell’ as well as EVICAN60 with 60 classes for 30 cell lines and their respective nuclei. Additionally, we provide three evaluation datasets accounting for varying image quality. As the dataset is only partially annotated, we encourage scientists and volunteers to add annotations and evaluate how the performance of classifiers changes.

Using the EVICAN2 dataset—with partially annotated images—in a pilot machine-learning application for cell and nucleus identification, we generated a classification and segmentation algorithm with an AP up to 61.6% at IoU scores above 0.5. This result demonstrates that training a classifier with our dataset of partially annotated and blurred background images was sufficient to segment cells and nuclei in the (fully annotated) evaluation images with reasonable performance. Other groups have reported more robust results (He *et al.*, 2017; Johnson, 2018). However, these algorithms rely on colored or stained images while EVICAN2 training produced a detection algorithm for unstained, grayscale images. Feature availability is reduced in grayscale images, as one channel is used instead of three, which partially explains the lower performance of resulting detection algorithms. Increasing the number of annotations is likely one way to improve the precision though we cannot speculate on the exact benefit.

The performance of our algorithm was best for the lowest difficulty images in our evaluation dataset. This can be explained with a higher degree of feature presentation in images with few cell–cell contacts, strong contrast (e.g. from PhC), high resolution and better-focused image conditions. The COCO dataset was designed with object types recognizable by a 4-year old (Lin *et al.*, 2014), while the EVICAN dataset includes cellular outlines and incorporated nuclei, that overlap, share a strong resemblance, and are often challenging to see without staining, even by a trained individual.

Better performance on high resolution and magnification images could arise from higher feature visibility that is lost in lower resolution. PhC images provide higher contrast; features appear more prominently, which facilitates feature detection in the convolutional process. Nevertheless, the limited dimensionality (as a consequence of the grayscale nature of the images) prevents the algorithm from searching for color-encoded features.

## 5 Conclusion

The proof-of-principle use of our dataset in the Mask-RCNN implementation performed adequately with our settings and computational resources, but we strongly encourage the scientific community to add further annotations or use more powerful computational tools to expand the capacity of resulting algorithms and increase segmentation accuracy. We believe that with the right tools (e.g. multi-GPU support) and advanced image augmentation, the EVICAN dataset, particularly the EVICAN60 version, can lead to transformative algorithm developments, similar to that seen in other computer vision fields. Such an algorithm, which is capable of not only segmenting cells and nuclei within an image but also discriminating among cell types in co-cultures would open the door to a new era of high-throughput cell microscopy. All cells in a microscopy field could be adequately measured label-free (i.e. quantification of cell spreading, elongation, circularity, etc.), multiple cell types in co-cultures could be instantly identified and changes in cell morphology could be e.g. linked to drug treatment or differentiation. We hope computer scientists and computational biologists use our dataset in efforts to achieve this goal.
